# Cross-Species, Amplifiable EST-SSR Markers for *Amentotaxus* Species Obtained by Next-Generation Sequencing

**DOI:** 10.3390/molecules21010067

**Published:** 2016-01-07

**Authors:** Chiuan-Yu Li, Tzen-Yuh Chiang, Yu-Chung Chiang, Hsin-Mei Hsu, Xue-Jun Ge, Chi-Chun Huang, Chaur-Tzuhn Chen, Kuo-Hsiang Hung

**Affiliations:** 1 Taiwan Endemic Species Research Institute, Nantou 552, Taiwan; snoopy60@gmail.com; 2 Graduate Institute of Bioresources, Pingtung University of Science and Technology, Pingtung 912, Taiwan; 3 Department of Life Sciences, National Cheng-Kung University, Tainan 701, Taiwan; tychiang@ncku.edu.tw; 4 Department of Biological Sciences, National Sun Yat-sen University, Kaohsiung 804, Taiwan; yuchung@mail.nsysu.edu.tw; 5 Department of Forestry, Pingtung University of Science and Technology, Pingtung 912, Taiwan; xinmeixu52@gmail.com (H.-M.H.); cct@mail.npust.edu.tw (C.-T.C.); 6 South China Botanical Garden, Chinese Academy of Sciences, Guangzhou 510650, China; xjge@scbg.ac.cn; 7 Kinmen National Park, Kinmen 892, Taiwan; hcc2889602@gmail.com

**Keywords:** *Amentotaxus*, endangered species, EST-SSR markers, genetic diversity, transcriptome

## Abstract

*Amentotaxus*, a genus of Taxaceae, is an ancient lineage with six relic and endangered species. Four *Amentotaxus* species, namely *A. argotaenia*, *A. formosana*, *A. yunnanensis*, and *A. poilanei*, are considered a species complex because of their morphological similarities. Small populations of these species are allopatrically distributed in Asian forests. However, only a few codominant markers have been developed and applied to study population genetic structure of these endangered species. In this study, we developed and characterized polymorphic expressed sequence tag-simple sequence repeats (EST-SSRs) from the transcriptome of *A. formosana*. We identified 4955 putative EST-SSRs from 68,281 unigenes as potential molecular markers. Twenty-six EST-SSRs were selected for estimating polymorphism and transferability among *Amentotaxus* species, of which 23 EST-SSRs were polymorphic within *Amentotaxus* species. Among these, the number of alleles ranged from 1–4, the polymorphism information content ranged from 0.000–0.692, and the observed and expected heterozygosity were 0.000–1.000 and 0.080–0.740, respectively. Population genetic structure analyses confirmed that *A. argotaenia* and *A. formosana* were separate species and *A. yunnanensis* and *A. poilanei* were the same species. These novel EST-SSRs can facilitate further population genetic structure research of *Amentotaxus* species.

## 1. Introduction

*Amentotaxus* (Taxaceae) represents an ancient evolutionary plant lineage and includes six relic species [1]. Currently, *Amentotaxus* species are locally distributed in Taiwan, from southwest China to Assam in the eastern Himalayas, and in southern Vietnam [2]. *Amentotaxus argotaenia* (Hance) Pilg., *A. formosana* H. L. Li, *A. poilanei* (Ferré & Rouane) D. K. Ferguson, and *A. yunnanensis* H. L. Li are considered a species complex; yet, the presence of intermediate morphotypes [3] poses some taxonomic challenges. While *A. argotaenia* and *A. yunnanensis* have been reported to have 2*n* = 36 chromosomes, the number of chromosomes in *A. formosana* and *A. poilanei* remains unknown [4]. There is little information about *Amentotaxus* genome size; only the nuclear DNA content of *A. yunnanensis* has been estimated as 2C = 60.40 [5]. In ecologic terms, these four species have a small population size and are allopatrically distributed in subtropical Asian forests. According to the conservation status assigned by the International Union for the Conservation of Nature [6], *A. argotaenia* is near threatened (NT), while *A. formosana*, *A. yunnanensis*, and *A. poilanei* are vulnerable (VU). Human overexploitation and development of the lumber industry have resulted in habitat loss and a continuous population decline of these four species. Therefore, governments must develop conservation strategies for providing a sustainable environment for the endangered *Amentotaxus* species. Population genetic structure studies have used inter-simple sequence repeat (ISSR), organellar DNA, and genomic SSR analyses to evaluate low genetic diversity within species and considerable genetic differentiation among populations or species [7,8]. Molecular markers are now widely employed for assessing genetic patterns or population genetic structure, and are vital for the management of threatened and endangered species. Such genetic information can facilitate the development of more efficient conservation strategies [9].

Expressed sequence tag-simple sequence repeats (EST-SSRs) differ from, and have several advantages over traditional genomic SSR markers, such as lower development cost and higher transferability across related species [10]. Moreover, the flanking sequences of EST-SSRs are located in well-conserved regions of transcribed genes from phylogenetically related species, permitting a high level of transferability [11,12,13]. EST-SSRs have been extensively used for quantifying genetic diversity and population genetic structure of plants [14]. While the use of microsatellites as species-specific markers is expensive, its cost effectiveness has benefited from the development of next-generation sequencing techniques. These enable the straightforward detection and characterization of SSR loci through highly *parallel sequencing processes*. Random sequencing approaches for identifying microsatellites are rapid and effective and can identify numerous useful and polymorphic microsatellites in unstudied or understudied species [15,16,17].

Although phylogeographic patterns of *Amentotaxus* species have already been assessed by ISSR, organellar DNA, and genomic SSR marker analyses [7,8], EST-SSR markers developed in this study provide an additional and distinct solution for investigating population genetic structure of *Amentotaxus* species. We applied a next-generation sequencing method for developing SSR markers from the transcriptome of *A. formosana*. Our objectives were as follows: (1) assess the frequency distribution of microsatellite motifs in the transcriptome of *A. formosana*; (2) examine the transferability of EST-SSR markers to three other *Amentotaxus* species; and (3) estimate the extent of genetic diversity and differentiation among *Amentotaxus* species using the developed EST-SSR markers. The ensuing genetic information can help develop conservation strategies for these threatened taxa.

## 2. Results and Discussion

### 2.1. Frequency Distribution of Various SSRs in the Transcriptome

**Figure 1:**
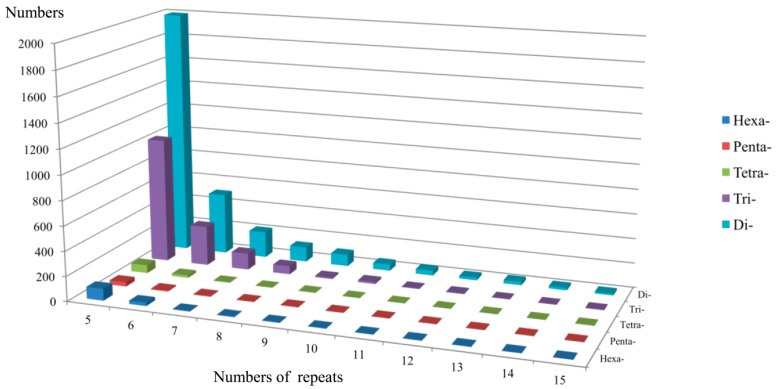
Frequency distributions of various *Amentotaxus formosana* EST-SSRs with different numbers of repeats.

The assembled unigenes from the transcriptome of *A. formosana* were screened for the presence of EST-SSRs, using the SSR Locator software. In total, 4955 putative EST-SSRs were identified from 68,281 unigenes (≥300 bp) with perfect di-, tri-, tetra-, penta-, and hexanucleotide motifs. Dinucleotide repeats constituted the largest group of repeat motifs, accounting for more than half of the total EST-SSR content (62.54%), followed by tri- (31.95%), hexa- (2.52%), tetra- (1.82%), and pentanucleotide (1.17%) repeats (Figure 1). Di- and trinucleotide repeats formed a large proportion of EST-SSRs, whereas the remaining repeats constituted 5.51% of EST-SSRs.

**Figure 2:**
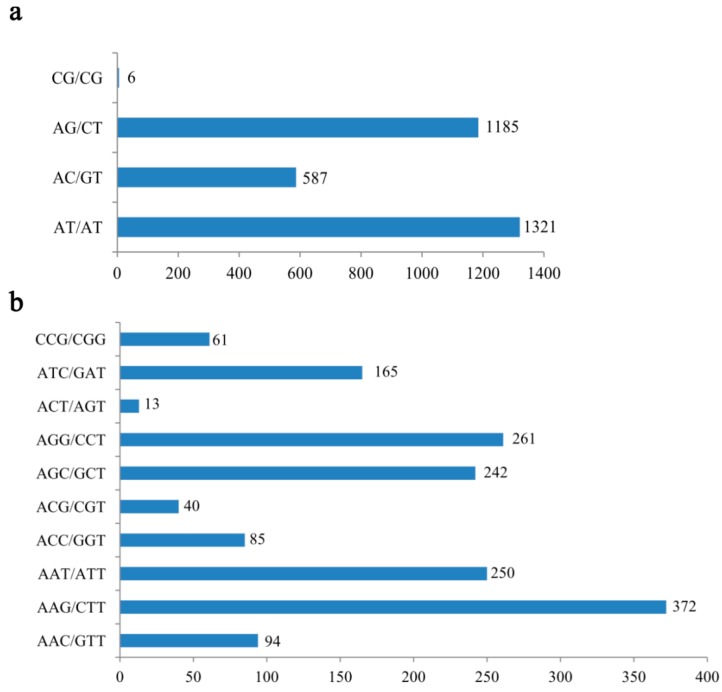
Number of various (**a**) di- and (**b**) trinucleotide repeat motifs in *Amentotaxus formosana*.

The number of EST-SSR repeats ranged widely from 5–15, 5–12, 5–9, 5–11, and 5–7 in di-, tri-, tetra-, penta-, and hexanucleotides, respectively. EST-SSR frequencies of dinucleotide repeats decreased stepwise with an increase in motif length. Among various SSRs, AT/AT accounted for the highest proportion (42.63%) of total dinucleotide repeats, followed by AG/CT (38.24%), AC/GT (18.94%), and CG/CG (0.19%) (Figure 2a). Among trinucleotide repeats, AAG/CTT was the most abundant (23.50%), followed by AGG/CCT (16.49%); while AAC/GTT, ACC/GGT, ACG/CGT, ACT/AGT, and CCG/CGG, accounted for <10% each (Figure 2b). By contrast, Ranade *et al.* [18] indicated the most abundant motif among angiosperms and gymnosperms was AG/CT. AT/AT is the most common repeat motif in other gymnosperms, such as *Pinus massoniana* [19], *P. dabeshanensis* [20], *P. densiflora* [21], and *Cryptomeria japonica* [22].

### 2.2. EST-SSR Marker Polymorphism and Cross-Species Transferability

Of the EST-SSR markers identified in the transcriptome of *A. formosana*, we selected 26 primer pairs to assess polymorphisms and transferability across *Amentotaxus* species (Table 1 and Table S1). All 26 primer pairs were successfully amplified to match the expected size using an initial repetitive DNA fragment. Twenty-three primer pairs were polymorphic among species. Amen24, Amen25, and Amen42 primer pairs were monomorphic in all four *Amentotaxus* species. The transferability of EST-SSR markers across *Amentotaxus* species was assessed by screening for the selected 26 EST-SSR markers. All 26 markers were transferable between *Amentotaxus* species. In total, 23 of the 26 EST-SSR markers were used to further analyze polymorphism information content (PIC), Hardy-Weinberg equilibrium (HWE), and genetic diversity of each *Amentotaxus* species. PIC values of the 23 polymorphic primer pairs ranged from 0.000 to 0.623 (mean = 0.169 ± 0.145) in *A. formosana.* PIC values were higher in *A. argotaenia* (mean = 0.328 ± 0.087) than in *A. poilanei* (mean = 0.291 ± 0.135) and *A. yunnanensis* (mean = 0.291 ± 0.184) (Table 1). In this study, transferability of polymorphic markers across *Amentotaxus* species was 100%. Furthermore, a high transferability of genomic SSRs was detected across *Amentotaxus* species [23,24]. The cross-amplification ratio varied among plants. Transferability was higher across *Amentotaxus* than *Taxus* [25] and *Pinus* [26] species, where it was 80% and 60%–80%, respectively. These results confirmed that the high cross-species transferability of the microsatellite markers developed in *A. formosana* can be used in related *Amentotaxus* species in order to assess genetic diversity and population genetic structure. The 23 polymorphic EST-SSR markers demonstrated low (PIC < 0.25) and moderate (0.5 > PIC > 0.25) polymorphisms in *A. formosana* and the other *Amentotaxus* species, respectively. A low EST-SSR polymorphism was also detected in *P. dabeshanensis* [20]. However, polymorphism of EST-SSRs was lower than that of genomic SSRs reported by Ko [24], indicating the occurrence of a typical low to medium polymorphism in the current study. Indeed, PIC values were 0.640, 0.614, 0.250, and 0.300 in *A. argotaenia*, *A. formosana*, *A. poilanei*, and *A. yunnanensis*, respectively [24]. The lower level of polymorphism of EST-SSRs compared to genomic SSRs may result from a higher level of conservation among expressed genes [27].

### 2.3. Genetic Diversity and Population Genetic Structure Analyses

Based on 23 polymorphic EST-SSRs, we assessed standard genetic diversity parameters, such as the number of alleles per locus (A), and observed (H_o_) and expected (H_e_) levels of heterozygosity, for each of the four taxa (Table 1). Overall, the number of alleles per locus ranged from 1–4 in *A. formosana* (mean = 1.783 ± 0.177), *A. argotaenia* (mean = 2.348 ± 0.198), and *A. yunnanensis* (mean = 2.478 ± 0.234), and from 1–3 in *A. poilanei* (mean = 2.174 ± 0.149). All 23 EST-SSRs were polymorphic in *A. argotaenia*; while 14, 17, and 19 EST-SSRs were polymorphic in *A. formosana*, *A. yunnanensis* and *A. poilanei*, respectively. H_o_ and H_e_ levels ranged between 0.000–0.667 and 0.000–0.678, respectively, in *A. formosana*; 0.000–0.909 and 0.080–0.670 in *A. argotaenia*; 0.000–0.917 and 0.000–0.740 in *A. yunnanensis*; and 0.000–1.000 and 0.000–0.653 in *A. poilanei.* The HWE test showed significant deviations for 11 and 10 EST-SSRs in *A. argotaenia*, and *A. formosana*, respectively; and 5 EST-SSRs in *A. yunnanensis* and *A. poilanei*. These could result from a deficiency of heterozygosity among *Amentotaxus* species.

**Figure 3:**
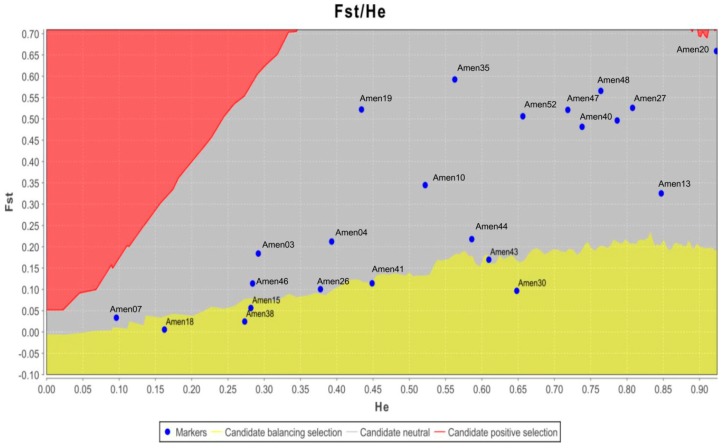
Assessment of outlier EST-SSR loci using LOSITAN software, which evaluated the expected distribution of the Wright inbreeding coefficient (F_st_) and expected heterozygosity (H_e_) by applying an island model of migration along with neutral markers. The five loci, Amen15, Amen18, Amen30, Amen38, and Amen43, were identified as outliers. Dots in the red and yellow areas represent candidate loci for positive or balanced selection.

The possibility of EST-SSR markers being located within functional genes, implies that the neutrality of EST-SSR markers observed in this study should not be assumed a priori. Non-neutral loci may bias population genetic structure analysis. Before proceeding any further, we used the LOSITAN software to identify possible outlier loci for all 23 polymorphic markers. Five outlier loci were identified (Figure 3): Amen15, Amen18, Amen30, Amen38, and Amen43. Because previous studies revealed that certain SSR markers were non-neutral, it is essential to conduct a neutrality test before applying SSR data to population genetic structure analysis [28,29,30]. Finally, we selected 18 polymorphic and neutral EST-SSR markers, with which to assess genetic diversity and population genetic structure among the four *Amentotaxus* species.

**Table 1:** Characterization of the selected 26 EST-SSRs from four *Amentotaxus* species.

	*A. argotaenia*					*A. formosana*					*A. yunnanensis*					*A. poilanei*				
Locus	A	H_o_	H_e_	PIC	HWE *p*-value	A	H_o_	H_e_	PIC	HWE *p*-value	A	H_o_	H_e_	PIC	HWE *p*-value	A	H_o_	H_e_	PIC	HWE *p*-value
Amen03	2.000	0.000	0.153	0.141	0.001	1.000	0.000	0.000	0.000	-	3.000	0.182	0.517	0.422	0.057	2.000	0.286	0.245	0.215	0.659
Amen04	2.000	0.167	0.500	0.375	0.021	1.000	0.000	0.000	0.000	-	3.000	0.417	0.344	0.307	0.842	2.000	0.429	0.337	0.280	0.471
Amen07	2.000	0.250	0.219	0.195	0.621	1.000	0.000	0.000	0.000	-	2.000	0.167	0.153	0.141	0.753	1.000	0.000	0.000	0.000	-
Amen10	2.000	0.167	0.375	0.305	0.054	1.000	0.000	0.000	0.000	-	2.000	0.100	0.375	0.305	0.020	2.000	0.833	0.486	0.368	0.080
Amen13	2.000	0.909	0.496	0.373	0.006	4.000	0.667	0.543	0.480	0.003	4.000	0.917	0.740	0.692	0.000	2.000	1.000	0.500	0.375	0.008
Amen15	3.000	0.500	0.406	0.371	0.721	1.000	0.000	0.000	0.000	-	2.000	0.500	0.375	0.305	0.248	3.000	0.286	0.255	0.240	0.978
Amen18	2.000	0.083	0.080	0.077	0.880	2.000	0.167	0.375	0.305	0.174	1.000	0.000	0.000	0.000	-	2.000	0.143	0.133	0.124	0.839
Amen19	3.000	0.333	0.500	0.449	0.000	2.000	0.000	0.219	0.195	0.005	1.000	0.000	0.000	0.000	-	1.000	0.000	0.000	0.000	-
Amen20	2.000	0.417	0.413	0.328	0.977	2.000	0.000	0.153	0.141	0.001	1.000	0.000	0.000	0.000	-	3.000	0.000	0.571	0.501	0.003
Amen24	1.000	0.000	0.000	0.000	-	1.000	0.000	0.000	0.000	-	1.000	0.000	0.000	0.000	-	1.000	0.000	0.000	0.000	-
Amen25	1.000	0.000	0.000	0.000	-	1.000	0.000	0.000	0.000	-	1.000	0.000	0.000	0.000	-	1.000	0.000	0.000	0.000	-
Amen26	2.000	0.333	0.278	0.239	0.488	2.000	0.083	0.080	0.077	0.880	3.000	0.583	0.434	0.369	0.565	3.000	0.857	0.541	0.453	0.268
Amen27	2.000	0.000	0.500	0.375	0.001	2.000	0.000	0.278	0.239	0.001	1.000	0.000	0.000	0.000	-	3.000	0.286	0.653	0.580	0.066
Amen30	4.000	0.750	0.670	0.606	0.562	2.000	0.000	0.494	0.372	0.003	4.000	0.500	0.618	0.562	0.174	3.000	0.429	0.449	0.406	0.497
Amen35	2.000	0.000	0.444	0.346	0.001	2.000	0.000	0.165	0.152	0.001	3.000	0.250	0.226	0.212	0.970	1.000	0.000	0.000	0.000	-
Amen38	2.000	0.091	0.351	0.290	0.014	2.000	0.000	0.298	0.253	0.001	1.000	0.000	0.000	0.000	-	3.000	0.429	0.357	0.325	0.914
Amen40	2.000	0.636	0.434	0.340	0.122	1.000	0.000	0.000	0.000	-	3.000	0.250	0.538	0.432	0.133	3.000	0.429	0.500	0.427	0.808
Amen41	3.000	0.667	0.486	0.424	0.392	1.000	0.000	0.000	0.000	-	4.000	0.714	0.541	0.502	0.904	2.000	0.857	0.490	0.370	0.047
Amen42	1.000	0.000	0.000	0.000	-	1.000	0.000	0.000	0.000	-	1.000	0.000	0.000	0.000	-	1.000	0.000	0.000	0.000	-
Amen43	4.000	0.500	0.535	0.498	0.075	2.000	0.100	0.255	0.222	0.055	3.000	0.545	0.533	0.432	0.838	3.000	0.714	0.602	0.523	0.214
Amen44	2.000	0.167	0.278	0.239	0.166	2.000	0.000	0.486	0.368	0.001	4.000	0.667	0.517	0.482	0.809	2.000	0.571	0.408	0.325	0.290
Amen46	2.000	0.083	0.469	0.359	0.004	1.000	0.000	0.000	0.000	-	2.000	0.182	0.165	0.152	0.740	2.000	0.429	0.337	0.280	0.471
Amen47	2.000	0.364	0.397	0.318	0.782	1.000	0.000	0.000	0.000	-	4.000	0.200	0.575	0.526	0.034	2.000	0.286	0.245	0.215	0.659
Amen48	2.000	0.000	0.500	0.375	0.001	2.000	0.200	0.180	0.164	0.725	1.000	0.000	0.000	0.000	-	2.000	0.000	0.490	0.370	0.008
Amen50	3.000	0.000	0.406	0.371	0.001	2.000	0.000	0.375	0.305	0.046	3.000	0.000	0.594	0.511	0.001	2.000	0.000	0.375	0.305	0.046
Amen52	2.000	0.000	0.165	0.152	0.001	4.000	0.000	0.678	0.623	0.000	2.000	0.000	0.444	0.346	0.001	1.000	0.000	0.000	0.000	-
Mean ^a^	2.348 ± 0.198	0.279 ± 0.057	0.394 ± 0.029	0.328 ± 0.087	-	1.783 ± 0.177	0.053 ± 0.030	0.1993 ± 0.044	0.1693 ± 0.145	-	2.478 ± 0.234	0.2683 ± 0.058	0.3343 ± 0.051	0.291 ± 0.184	-	2.174 ± 0.149	0.359 ± 0.067	0.347 ± 0.043	0.291 ± 0.135	-
Mean ^b^	2.167 ± 0.090	0.250 ± 0.064	0.370 ± 0.028	0.317 ± 0.070	-	1.778 ± 0.222	0.053 ± 0.038	0.175 ± 0.051	0.152 ± 0.145	-	2.556 ± 0.258	0.258 ± 0.067	0.342 ± 0.057	0.300 ± 0.177	-	2.000 ± 0.162	0.348 ± 0.083	0.343 ± 0.051	0.281 ± 0.140	-

^a^ for 23 polymorphic EST-SSRs; ^b^ for 18 polymorphic and neutral EST-SSRs; HWE: Hardy-Weinberg equilibrium.

The number of alleles per locus for the 18 polymorphic and neutral EST-SSRs was highest in *A. yunnanensis* (mean = 2.556 ± 0.258) and lowest in *A. formosana* (mean = 1.778 ± 0.222). In addition, the level of observed heterozygosity was highest in *A. poilanei* (mean= 0.348 ± 0.083) and lowest in *A. formosana* (mean = 0.053 ± 0.038). The levels of observed heterozygosity in all species were significantly lower than expected heterozygosity levels, and lower than those observed in other conifers [31,32], suggesting a deficiency of heterozygosity in *Amentotaxus* species. Generally, widespread species exhibit higher levels of genetic diversity than narrowly distributed species. The patterns of genetic variation are attributed to numerous evolutionary factors, such as founder effects, bottlenecks, and gene flows. Therefore, current population size may not be a reliable indicator for determining genetic diversity levels [33,34,35,36]. Nevertheless, Ge *et al.* [8] reported high levels of genetic diversity in *A. poilanei* and *A. yunnanensis* with small population sizes. Our results agree with other studies reporting high genetic diversity levels in a few threatened and endangered species, with a small population size and a narrow distribution [37,38,39].

**Figure 4:**
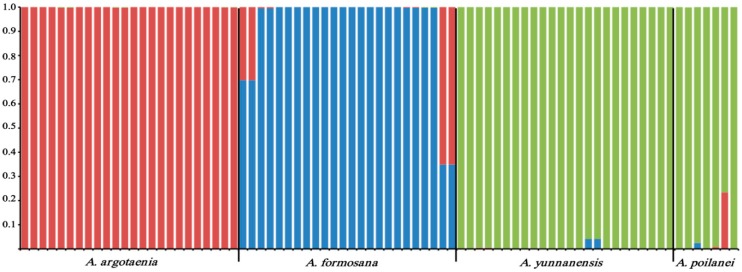
Genetic composition of *Amentotaxus* species in three clusters (K = 3) were detected through structure analyses by using the highest ΔK value.

We also examined population genetic structure at a finer resolution using STRUCTURE software [40]. Here, ΔK values computed for all classes indicated a strong signal for K = 3 (ΔK = 2.754). The proportions of each individual in each population were assigned to three clusters. For K = 3, the four *Amentotaxus* species were divided into three clusters, with *A. yunnanensis* and *A. poilanei* clustering together. Several individuals displayed an intermixed composition, which could be attributed to a historical gene flow between species (Figure 4). Furthermore, pairwise comparisons indicated significant genetic differentiation (F_st_) between species (F_st_ = 0.134–0.315; *p* < 0.05). Consistent with STRUCTURE analysis results, *A. formosana* exhibited high genetic differentiation levels (F_st_ = 0.242–0.315), compared to the closely related *A. yunnanensis* and *A. poilanei* (F_st_ = 0.134) species, because of geographical isolation. Similar trends were observed by organellar DNA, genomic SSR, and ISSR fingerprinting analysis [7,8], confirming that both, *A. argotaenia* and *A. formosana*, could be considered as separate species, and *A. yunnanensis* and *A. poilanei* as the same species.

## 3. Experimental Section

### 3.1. Plant Material and DNA Extraction

Twenty-four plant samples of *A. formosana* were collected from Chinshuiying (120°46′ E, 22°16′ N), Taiwan. In addition, we sampled 24 individuals of *A. argotaenia* from Suining, Hunan Province (110°10′ E, 26°29′ N, *N* = 12), and Datian, Fujian Province (117°48′ E, 25°40′ N, *N* = 12), China; 24 individuals of *A. yunnanensis* from Malipo (104°58′ E, 23°16′ N, *N* = 12) and Xichou (104°13′ E, 23°16′ N, *N* = 12), Yunnan Province, China; and seven individuals of *A. poilanei* from Lao Va Chai Municipality (105°04′ E, 23°06′ N), Vietnam. Samples for DNA extraction were dried in silica gel. Total genomic DNA was extracted using cetyltrimethylammonium bromide [41].

### 3.2. cDNA Preparation, Illumina Sequencing, and de Novo Assembly

Trizol reagent (Life Technologies, Carlsbad, CA, USA) was used for extracting total RNA from the leaves of whole young juveniles of *A. formosana* previously frozen in liquid nitrogen and ground into a powder. Total extracted RNA was purified using the RNeasy Mini RNA kit (QIAGEN, Hilden, Germany) according to the manufacturer’s instructions. mRNA was isolated from purified total RNA using Dynabeads (Life Technologies). The purity and quality of mRNA were verified. A cDNA library was constructed using the SMART™ cDNA library construction kit (Clontech, Mountain View, CA, USA). The synthesized cDNA was subjected to end-repair and phosphorylation, in which the repaired cDNA fragments were 3′-adenylated using Klenow 3′ to 5′ exopolymerase, and then ligated with an adapter using T4 DNA ligase. Finally, the cDNA library was constructed using a 200 bp insertion fragment excised from the gel. PCR primer pairs PE 1.0 and 2.0 (Illumina Inc., San Diego, CA, USA) were used for amplifying the cDNA fragments. The cDNA library was sequenced using the Illumina HiSeq™ 2000 (Illumina Inc.).

Quality trimming was performed to filter out poor *quality* or ambiguous data from raw sequencing reads. The reads from >10% of bases gave a poor quality score (Q < 20), and ambiguous sequences with an excess of N nucleotides were removed. We then applied SOAPdenovo software (http://soap.genomics.org.cn/soapdenovo.html) for a *de novo* assembly of the transcriptome using default settings, except for the K-mer value (default K = 29) [42]. To this end, we applied de Bruijn graphs to assemble the reads assigned to each gap and construct contigs without ambiguous bases. Paired-end libraries *and connecting overlapping contigs were employed* to build the scaffolds. Paired-end reads were used to fill in gaps and generate larger scaffolds, which were defined as unigenes. Finally, we used overlapping unigenes to assemble a large and continuous DNA sequence and screen for EST-SSR markers.

### 3.3. Detection of EST-SSR Markers and Primer Design

SSR Locator software [43] was applied to screen for SSR regions within 68,281 unigenes (>300 bp). Parameters were adjusted to identify the perfect di-, tri-, tetra-, penta-, and hexanucleotide motifs with a minimum of five and a maximum of 15 repeats. Next, 26 microsatellites were selected to determine transferability across species. We assumed that tri- or hexanucleotide *motifs* might possess a higher level of *polymorphism* than the other *motifs*. Therefore, most of the selected 26 microsatellites contained tri- or hexanucleotide *motifs*. EST-SSR primers were designed using Primer3 software [44], and primer sequences of the identified microsatellites are listed in Table S1.

### 3.4. DNA Amplification and Genotyping

The 26 selected microsatellites were examined for transferability and polymorphism among species. Microsatellites were subjected to PCR amplification in a 25-µL volume containing 10 ng genomic DNA, 0.2 mM dNTP, 2 mM MgCl_2_, and 5 pmol of each primer. Forward primers used for the PCR reaction were fluorescently labeled. PCR conditions were as follows: 3 min at 94 °C, 40 cycles of 30 s at 94 °C, 30 s at a primer-specific annealing temperature (Table S1), 30 s at 72 °C, and a final extension step of 5 min at 72 °C. PCR products were separated using an ABI 3100 automated sequencer, and fragment size was assessed using genemapper Version 3.7 software (Applied Biosystems, Foster City, CA, USA).

### 3.5. Data Analysis

After assessing transferability across species, standard genetic diversity parameters, A, H_o_, and H_e_, were calculated for polymorphic microsatellites using GenAlEx Version 6.5 software [45]. In addition, we conducted PIC and HWE tests on PowerMarker Version 3.25 [46] and GENEPOP Version 3.4 software [47]. A biased genetic diversity analysis may have resulted because of non-neutral loci. A pairwise comparison of the genetic differentiation (F_st_) outlier test was performed using LOSITAN software [48,49] in order to identify candidate non-neutral SSR loci, which might have biased the genetic diversity analysis. The identified *neutral* SSR loci were used for *further* genetic diversity and population genetic analyses of each species. F_st_ among species was performed using GenAlEx Version 6.5 software [45]. Genetic composition of *Amentotaxus* species was examined using STRUCTURE Version 2.3.3 software [40,50,51]. structure applies a Bayesian method for inferring K values, without using prior information of individual sampling locations. Stability of the results was estimated by executing 10 separate runs with K = 1–5. Each run was pursued for 1,000,000 Markov chain Monte Carlo interactions with an initial burn-in of 100,000 and an ancestry model that allowed for admixture [52]. The most favorable grouping number (K) was determined by calculating ΔK [53] using STRUCTURE HARVESTER Version 0.6.8 software [54].

## 4. Conclusions

A total of 4955 EST-SSR markers were revealed when screening the transcriptome of *A. formosana*. The AT/AT motif was the most common repeat unit in *A. formosana* and other gymnosperms, such as *P. massoniana*, *P. dabeshanensis*, *P. densiflora*, *P. dabeshanensis*, and *C. japonica*. The identified polymorphic and neutral EST-SSR markers were used to assess genetic diversity and differentiation among *Amentotaxus* species. Population genetic structure analysis indicated that *A. argotaenia* and *A. formosana* were separate species and that *A. yunnanensis* and *A. poilanei* were the same species. In summary, the *Amentotaxus* species exhibited a higher level of genetic differentiation, except between *A. yunnanensis* and *A. poilanei*. The low level of genetic diversity estimated by EST-SSR markers is possibly caused by past fragmentation and recent habitat degradation. EST-SSR markers developed in this study can potentially be used for assessing genetic diversity and population genetic structure among *Amentotaxus* species.

## Supplementary Materials

Supplementary materials can be accessed at: http://www.mdpi.com/1420-3049/21/1/67/s1.
