# Supplementary material for: Cross-Species, Amplifiable EST-SSR Markers for Amentotaxus Species Obtained by Next-Generation Sequencing
Source: Molecules. 2016 Jan 7;21(1):67. doi: 10.3390/molecules21010067 (PMC6273106; doi:10.3390/molecules21010067)
Supplement: Supplementary file 1 [file molecules-21-00067-s001.pdf]

Supplementary Materials: Cross-Species, Amplifiable EST-SSR Markers for *Amentotaxus* Species Obtained by Next-Generation Sequencing

Chiuan-Yu Li [1,2,†], Tzen-Yuh Chiang [3,†], Yu-Chung Chiang [4,†], Hsin-Mei Hsu [5], Xue-Jun Ge [6], Chi-Chun Huang [7], Chaur-Tzuhn Chen [5] a nd Kuo-Hsiang Hung [2,*]

Table S1. Characteristics and functional annotations of 26 microsatellite primers were developed in *Amentotaxus formosana.*

Locus	Repeat Motif	Primer Sequence(5′–3′)	Allele Size (bps)	*Ta*				Cross Amplification	Accession Number	BLAST Top Hit Acc. No.	Description	*e*-Value
				Ar	Fo	Yu	Po					
Amen03	$(TGTTGC)_4$ $(GTT)_4$	F: CATTCTGATTAGTTTGCTTC<br>R:TAATGGTCAATCTGCTAGTC	179–187	50	50	50	50	4/4	LN907616	XP_002269363.2	PREDICTED: *Vitis vinifera* hypothetical protein	0
Amen04	$(GGAATG)_6$	F:GAGAACATTACAGGAAGTCA<br>R:AGGCATAATTGAACTGATT	178–196	50	50	50	48	4/4	LN907617	XP_001757450.1	*Physcomitrella patens* subsp. *patens* predicted protein	4.77×10^{-41}
Amen07	$(ATTTT)_3$ $(ATTTTTT)_3$	F:CATGGAGAGTCCTGATAGTA<br>R:CTCATACGGTCTGTAGAAAA	203–208	54	54	54	54	4/4	LN907618	ACN26960.1	*Zea mays* unknown protein	2.92×10^{-62}
Amen10	$(CCA)_4$ $(CCT)_{12}$	F:CATAGCACTTGAGATCACTT<br>R:GCATTTATAGAGTTTGACGA	349–358	50	50	50	50	4/4	LN907619	XP_002534324.1	*Ricinus communis* putative arginine/serine-rich splicing factor,	6.90×10^{-15}
Amen13	$(CTATCC)_4$	F:ATATCACTGGCAGTCTCTT<br>R:ATATCTATATGGTTGGCCTC	254–290	50	50	50	50	4/4	LN907620	CAN74005.1	*Vitis vinifera* hypothetical protein VITISV_006236	4.54×10^{-17}
Amen15	$(CTG)_3$ $(GATTGG)_4$	F:CTATTGTCAGAGAAATCGAA<br>R:GAAAGAGAAAGAGAAAGTGG	264–279	53	53	53	53	4/4	LN907621	XP_002265605.1	*Vitis vinifera* PREDICTED: partial hypothetical protein	1.22×10^{-41}
Amen18	$(AAGTGG)_4$	F:CATCTCGCTTTCTATTCAT<br>R:AAAGCTTTATTGAACTTCG	273–279	48	48	48	48	4/4	LN907622	None	None	None
Amen19	$(TGATGG)_4$	F:TAGGACAAAGAAGATAGCAG<br>R:CATCAACACCATCACTGT	245–257	50	50	50	50	4/4	LN907623	XP_002285304.1	*Vitis vinifera* PREDICTED: hypothetical protein	1.45×10^{-56}

Table S1. *Cont.*

Locus	Repeat Motif	Primer Sequence(5′–3′)	Allele Size (bps)	*Ta*				Cross Amplification	Accession Number	BLAST Top Hit Acc. No.	Description	*e*-Value
				Ar	Fo	Yu	Po					
Amen20	(CGGGAT)$_4$ N(GAGACG)$_3$N (GGAAGG)$_4$	F:AAATCCTAGAAACGGTATTT<br>R:TTGTCTCAGCAGTTTCTTAT	279–300	50	50	50	50	4/4	LN907624	None	None	None
Amen24	(TCCTGC)$_6$	F:CTCTTTATAACAGCCTCCTC<br>R:GAAGGCTTCTATTTCTCTGT	206	51	51	51	51	4/4	LN907625	XP_002525143.1	*Ricinus communis* conserved hypothetical protein	3.49×10^{-19}
Amen25	(CCT)$_7$	F:GAATCTACTCAATGTATGCG<br>R:GAGTAGAGACGTTCCAGTG	236	49	49	49	49	4/4	LN907626	XP_002518826.1	*Ricinus communis* conserved hypothetical protein	0
Amen26	(AGGAAG)$_6$	F:TATCTAGAGTGCTGTGTTGG<br>R:TTGAATTAGCATTAATAGGG	239–251	52	52	52	52	4/4	LN907627	ABK24854.1	*Picea sitchensis* unknown protein	1.07×10^{-96}
Amen27	(AGGATT)$_4$	F:TGTATGGATTTAAATAGGGA<br>R:AATCCTAATCCTAACCCTAA	164–172	50	50	50	50	4/4	LN907628	XP_002973804.1	*Selaginella moellendorffii* hypothetical protein SELMODRAFT_100054	2.10×10^{-146}
Amen30	(CTGCCC)$_4$	F:GCATAGGTGTAGCTATGAAC<br>R:ATACTTGTGCAGTTTGATTT	402–414	50	50	50	50	4/4	LN907629	XP_002300693.1	*Populus trichocarpa* predicted protein	1.62×10^{-58}
Amen35	(GAT)$_{10}$	F:CATTGGGCAAATTCAGTGTC<br>R:GCCTCCCACCCATTAGTAGA	237–252	50	50	50	50	4/4	LN907630	XP_002268183.1	*Vitis vinifera* PREDICTED: hypothetical protein	0
Amen38	(CCA)$_9$	F:AAGAAATCTGGGGCACTGGA<br>R:TGCACATCTTCTTCGTCACA	119–137	50	50	50	50	4/4	LN907631	ADE77044.1	*Picea sitchensis* unknown protein	0
Amen40	(CA)$_{12}$	F:ACCCAGCTCAATACCAAAGC<br>R:GCGAAATGTTGGTGACAGAC	350–380	51	51	51	51	4/4	LN907632	None	None	None
Amen41	(AGAA)$_6$	F:CATGCAAATTGAGGCCAGTA<br>R:ATAGGAGGAGAGGGCAAGGT	146–158	50	50	50	50	4/4	LN907633	EEC84662.1	*Oryza sativa* Indica Group hypothetical protein OsI_31560	5.08×10^{-10}
Amen42	(AGA)$_9$	F:TGATGAGGAAGATGGGATGA<br>R:CTTGGAGCATTCTCTGATGC	206	50	50	50	50	4/4	LN907634	XP_002523095.1	*Ricinus communis* putative U3 small nucleolar RNA-associated protein	1.25×10^{-22}

Table S1. *Cont.*

Locus	Repeat Motif	Primer Sequence(5′–3′)	Allele Size (bps)	*Ta*				Cross Amplification	Accession Number	BLAST Top Hit Acc. No.	Description	*e*-Value
				Ar	Fo	Yu	Po					
Amen43	$(TGG)_{10}$	F:AAGGTCTGTTGAAGTTTCGC<br>R:AAGGGAGCGATTATTGGTTG	180–210	50	50	50	50	4/4	LN907635	XP_002969636.1	*Selaginella moellendorffii* hypothetical protein SELMODRAFT_231344	5.07×10^{-136}
Amen44	$(GGAGA)_4$	F:GAGCACGTGGGGTTCTATG<br>R:GAGCGAGGGCAATAAAAGAG	95–100	50	50	50	50	4/4	LN907636	ABK23596.1	*Picea sitchensis* unknown protein	1.07×10^{-68}
Amen46	$(AGTTG)_6$	F:GTGTATAGGCCGCACCTTTT<br>R:TGCACTTGCAACAACACAAC	169–174	50	50	50	50	4/4	LN907637	CAN59753.1	*Vitis vinifera* hypothetical protein VITISV_036639	6.37×10^{-178}
Amen47	$(CTTCC)_8$	F:GGAAACTGGAATAGTCGGGA<br>R:ATTTGCTGCAACAGAGCAAG	148–203	50	50	50	50	4/4	LN907638	ABK23165.1	*Picea sitchensis* unknown protein	8.39×10^{-54}
Amen48	$(CT)_{15}$	F:ATTTTATGGGCAGTCAAGGC<br>R:TAGAGGGAGAAGGAGACGGA	237–267	52	52	52	52	4/4	LN907639	None	None	None
Amen50	$(ATAGA)_{10}$	F:CATAGACTACGCTCACCCCA<br>R:CTCCCCTACCTACCCTACCC	220–260	53	53	53	53	4/4	LN907640	None	None	None
Amen52	$(AATAG)_8$	F:TAGGGCATACCTATCTGCCA<br>R:GCCTCCCAGAGCCATAGATA	288–318	50	50	50	50	4/4	LN907641	None	None	None

The forward (F) and reverse (R) sequences, repeat motif, allele size (bps), annealing temperature when run individually (*Ta*), cross amplification, the GenBank accession number and Blast analysis are indicated. Abbreviations Ar, Fo, Yu, and Po indicate *A. argotaenia*, *A. formosana*, *A. yunnanensis*, and *A. poilanei*, respectively.
